# Calls to action on lung cancer management and research

**DOI:** 10.1093/oncolo/oyae169

**Published:** 2024-07-13

**Authors:** May-Lucie Meyer, Fred R Hirsch, Paul A Bunn, Peter Ujhazy, David Fredrickson, Christine D Berg, David P Carbone, Balazs Halmos, Harpreet Singh, Hossein Borghaei, Andrea Ferris, Corey Langer, Sanja Dacic, Tony S Mok, Solange Peters, Bruce E Johnson

**Affiliations:** Hematology and Oncology Department, Tisch Cancer Institute at Mount Sinai, Icahn School of Medicine and Thoracic Oncology Center, New York, NY, United States; Hematology and Oncology Department, Tisch Cancer Institute at Mount Sinai, Icahn School of Medicine and Thoracic Oncology Center, New York, NY, United States; Division of Medical Oncology, University of Colorado School of Medicine, Aurora, CO, United States; Translational Research Program, Division of Cancer Treatment and Diagnosis, National Cancer Institute, Rockville, MD, United States; Astra Zeneca, Washington, DC, United States; Early Cancer Detection Consultant, Bethesda, MD, United States; Division of Medical Oncology, The Ohio State University—James Comprehensive Cancer Center, Columbus, OH, United States; Department of Oncology, Montefiore Medical Center, Albert Einstein College of Medicine, Bronx, NY, United States; US Food and Drug Administration (FDA), Washington, DC, United States; Fox Chase Cancer Center, Philadelphia, PA, United States; LUNGevity Foundation, Chicago, IL, United States; Abramson Cancer Center, Perelman School of Medicine, University of Pennsylvania, Philadelphia, PA, United States; Department of Pathology, Yale School of Medicine, New Haven, CT, United States; State Laboratory of Translational Oncology, The Chinese University of Hong Kong, Hong Kong, People’s Republic of China; Department of Oncology, University Hospital CHUV, Lausanne, Switzerland; Department of Medical Oncology, Dana-Farber Cancer Institute, Harvard Medical School, Boston, MA, United States

**Keywords:** screening, smoking cessation, non–small cell lung cancer, small cell lung cancer, immunotherapy, biomarkers

## Abstract

Lung cancer, the leading cause of cancer-related deaths globally, remains a pressing health issue despite significant medical advances. The New York Lung Cancer Foundation brought together experts from academia, the pharmaceutical and biotech industries as well as organizational leaders and patient advocates, to thoroughly examine the current state of lung cancer diagnosis, treatment, and research. The goal was to identify areas where our understanding is incomplete and to develop collaborative public health and scientific strategies to generate better patient outcomes, as highlighted in our “Calls to Action.” The consortium prioritized 8 different calls to action. These include (1) develop strategies to cure more patients with early-stage lung cancer, (2) investigate carcinogenesis leading to lung cancers in patients without a history of smoking, (3) harness precision medicine for disease interception and prevention, (4) implement solutions to deliver prevention measures and effective therapies to individuals in under-resourced countries, (5) facilitate collaborations with industry to collect and share data and samples, (6) create and maintain open access to big data repositories, (7) develop new immunotherapeutic agents for lung cancer treatment and prevention, and (8) invest in research in both the academic and community settings. These calls to action provide guidance to representatives from academia, the pharmaceutical and biotech industries, organizational and regulatory leaders, and patient advocates to guide ongoing and planned initiatives.

## Teaser

The New York Lung Cancer Foundation brought together experts from academia, the pharmaceutical and biotech industries, as well as organizational leaders and patient advocates, to examine the current state of lung cancer diagnosis, treatment, and research. The resulting Calls to Action included 8 main points, described here.

## Introduction

To address critical issues needed to improve outcomes for patients at risk for and with lung cancer, the New York Lung Cancer Summit gathered global experts from academia, the Food and Drug Administration (FDA), the National Cancer Institute (NCI), pharmaceutical and biotech industries, and patient advocacy groups. The summit aimed to (1) review the current state of lung cancer management and research, (2) identify existing gaps, (3) explore collaborative solutions, and (4) recommend steps to advance the field. The summit included insights from the US, Europe, Asia, and lower-income countries, offering a comprehensive global view. The participants proposed “Calls to Action” which are summarized in the abstract and later in the manuscript.

## Strategies of prevention and early detection

### Prevention

Although lung cancer deaths have decreased in recent years, they remain the most common cause of cancer-related deaths globally.^1^ This decline is attributed to reduced rates of tobacco smoking; earlier disease detection; advances in surgical and radiation techniques; and advancements in medical treatments.^2^

Tobacco smoking is the leading risk factor for lung cancer.^3^ Unfortunately, while smoking cessation programs are recommended, they are not standardized in clinical care.^3^ Additionally, popular alternatives such as cannabis smoking and vaping, especially among youth, present additional less defined lung cancer risks.^4,5^ Air pollution, family history, DNA repair capabilities, and germline mutations also contribute to lung cancer.^6,7^ Inflammatory responses triggered by fine particulate matter (PM2.5) are associated with EGFR- and KRAS-driven lung cancers.^7^

Preventing smoking initiation and encouraging cessation are crucial, as robust prevention and cessation efforts give rise to lower lung cancer rates and mortality.^8^ Clinicians should directly refer patients to cessation programs, combining behavioral counseling with pharmacotherapy, with enhanced accessibility and affordability.^9^ Enforcing age restrictions, advertising bans, and taxing tobacco products can further discourage smoking. The European Union has banned flavored combustible tobacco products to reduce their appeal to young people.^10^

### Early detection

Risk-prediction models have shown that lung cancer screening can significantly prolong life expectancy.^11^ Both the NLST and the NELSON trials have reported a 20% decrease in lung cancer mortality among the at-risk population through low-dose computerized tomography (LDCT) screening,^11^ with a cost-effectiveness ranging from $27 756 to $243 077 per quality-adjusted life year.^11^ This has led to recommendations for lung cancer screening in high-risk groups in the United States.^11^ However, global screening rates remain low.^12^ Limitations include misunderstandings about its benefits, medical mistrust, and media biases. Strengthening ties with community organizations and tailoring educational messages to cultural contexts could improve screening rates. For example, Dr. Ola Khorshid reported Egypt employs mobile CT scans to screen high-risk patients who have limited access to major health care centers.

The American Cancer Society guidelines recently expanded its screening guidelines to include individuals aged 50-80 with a 20-pack-year smoking history.^13^ Discussions persist on expanding these criteria further. A Taiwanese study proposes a screening approach considering factors including family history, passive smoking, lung disease history, and household exposures.^14^ Internationally, successful initiatives provide examples for policy development. Pulmonary nodule clinics, often in academic settings, are growing in number to improve nodule assessment and clinical follow-up.^15^

**Figure 1.**
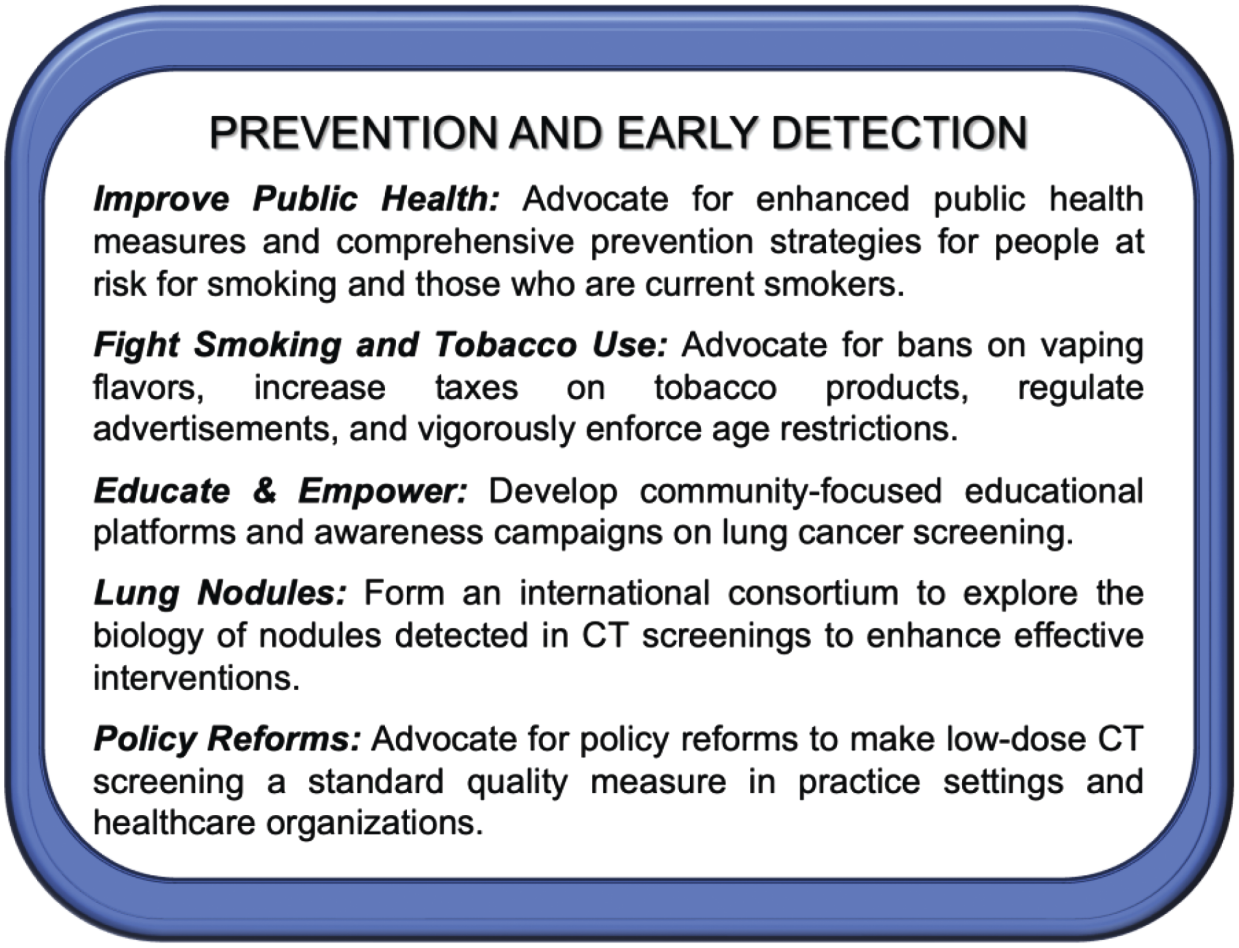
2023 New York Lung Cancer Foundation Consensus Summit Calls to Action—strategies of prevention and early detection.

Calls to Action are shown in Figure 1.

## Non–small cell lung cancer treatment

**Figure 2.**
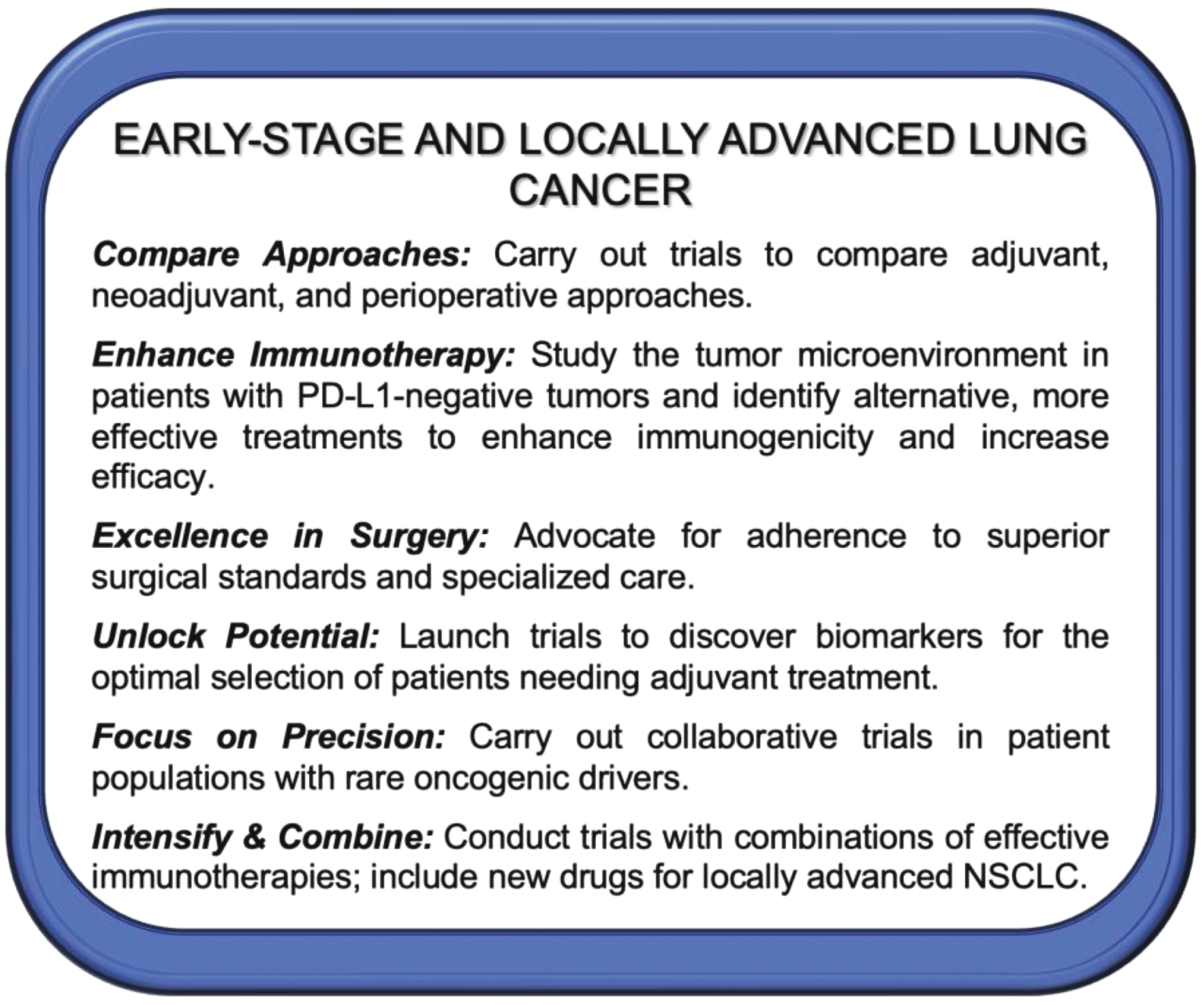
2023 New York Lung Cancer Foundation Consensus Summit Calls to Action—strategies for early-stage and locally advanced lung cancer.

**Figure 3.**
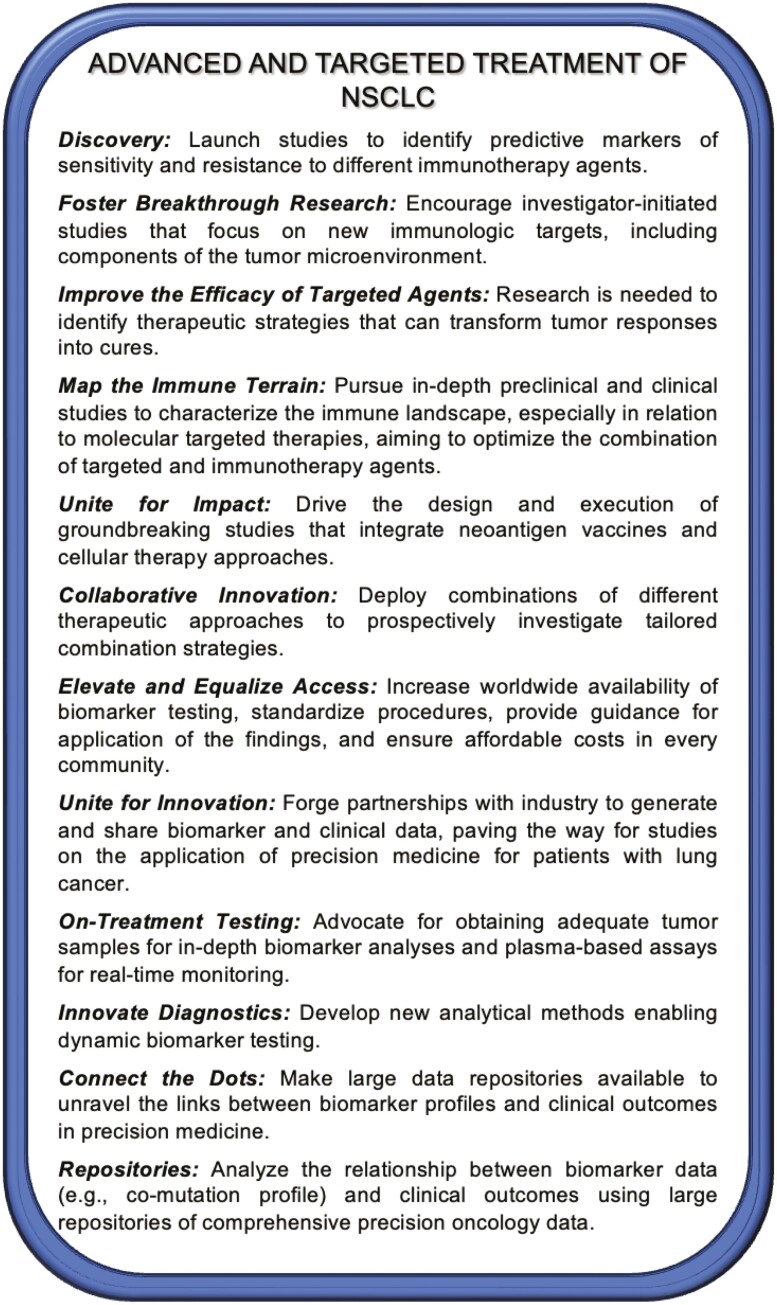
2023 New York Lung Cancer Foundation Consensus Summit Calls to Action—strategies for advanced-stage NSCLC and targeted treatment.

For patients with non–small cell lung cancer (NSCLC) without oncogenic drivers, immune checkpoint inhibitors (ICIs) have transformed the treatment landscape.^16^ Uncertainties remain, including the best sequence of agents, their application in patients with oncogene-driven tumors, and the duration of therapy. Key recommendations for future clinical trials are listed in Figures 2 and 3.

### Perioperative, neoadjuvant, and adjuvant therapy in early-stage NSCLC

ICIs for early-stage NSCLC have been explored through neoadjuvant, adjuvant, and perioperative strategies. Two notable phase 3 trials, IMpower 010 and KEYNOTE-091/PEARLS, showed prolonged event-free survival (EFS) with adjuvant atezolizumab and pembrolizumab, respectively.^17^ CheckMate-816 demonstrated the benefit of neoadjuvant nivolumab plus chemotherapy versus chemotherapy alone in patients with resectable NSCLC (stage IB-IIIA).^17^ The AEGEAN perioperative study of durvalumab showed significant increases in pathologic complete responses and EFS.^17^ The KEYNOTE-671 trial was the first to report an overall survival benefit with perioperative pembrolizumab.^17^ Checkmate 77T and NEOTORCH, have also shown improved EFS with perioperative ICIs.^17^ These approaches (neoadjuvant, perioperative, and adjuvant) have not been directly compared, and practices vary by treatment center and country. Patients with small, negative PD-L1 tumors have limited benefit, highlighting the need for better treatment strategies. Access to high-quality multidisciplinary care and surgical expertise is also vital.

### Oncogenic alterations

In patients with resectable or potentially resectable *EGFR*-mutant NSCLC, neoadjuvant treatment with tyrosine kinase inhibitors (TKIs) has been suggested, and a phase 3 study is ongoing.^18^ Adjuvant osimertinib showed dramatic survival benefits in the ADAURA trial, reducing the risk of death and brain recurrences by 50%, and making it a new standard for patients with resected stage IB-IIIA NSCLC.^19^

For patients with *ALK* rearrangements, the recent ALINA study has reported a clear EFS benefit with adjuvant alectinib.^20^ Treatment strategies for patients with early-stage NSCLC harboring rarer oncogenic alterations are less defined, as these often represent small subgroups in studies.^17^ Conducting large trials in small populations is challenging; therefore, if smaller cohorts show benefit, these should be considered in drug approvals.

### Locally advanced NSCLC

The standard of care for patients with locally advanced NSCLC has improved, especially with the integration of immunotherapy. The PACIFIC study was a landmark study demonstrating that consolidation with durvalumab in patients with unresectable stage III NSCLC treated with combined modality therapy who had not progressed resulted in a 5-year survival benefit of 47.5 months, versus 29.1 months with placebo.^21^ Ongoing studies are exploring additional immunotherapies, including nivolumab and ipilimumab, combinations with PARP inhibitors or radiotherapy.^22-25^

Calls to Action are shown in Figure 2.

### Advanced NSCLC

ICIs revolutionized the treatment of advanced NSCLC, and are now an integral part of the first-line treatment.^16^ These include monotherapies with pembrolizumab, atezolizumab, and cemiplimab for tumors selected for high PD-L1 expression as well as combinations with chemotherapy for patients with PD-L1-low/negative tumors.^16,26^ Additionally, combinations of ICIs (like nivolumab plus ipilimumab and durvalumab plus tremelimumab) have been explored, alone or with chemotherapy, showing increased efficacy for some patients (eg, with PD-L1-negative, *STK11/KEAP1*-mutated tumors).^27^ New targets now being studied include lymphocyte activation gene-3 (LAG-3), T-cell immunoglobulin and mucin-domain-containing-3 (TIM-3), and T-cell immunoreceptor with ITIM domain (TIGIT).^28^ Key questions (summarized in Table 1) persist regarding which patients will benefit most, the best treatment length, order of therapies, and how to effectively combine ICIs with targeted treatments.

**Table 1.** Key questions that can and need to be addressed through future clinical trial designs.

To address this clinical question…	Clinical trials need to…
Biology of immunotherapy treatment	
Why do some tumors not react to neoadjuvant therapy?	Examine cellular composition of tumors that do not respond to neoadjuvant therapy.
How do different lung cancer tumors evolve over time?	Characterize different biomarkers for both early and advanced metastatic stages in resident immune cells.
Response to therapy	
Why do some tumors with targetable molecular alterations fail to show response to immunotherapy?	Retrospectively correlate molecular signatures and response to immunotherapy with clinical outcomes.
Is adjuvant therapy necessary in some patient groups?	Examine responses to neoadjuvant with or without adjuvant therapy in different patient populations.
In what combinations is adjuvant therapy most effective?	Study adjuvant immunotherapy alone vs. in combination.
Disease recurrence	
Is treating until disease progression or toxicity the best approach to delay relapse?	Examine response after different durations of adjuvant treatment.
Do persisting tumors present novel targets for combination therapy?	Study different combination therapies in relapsed populations.

### Targeted therapy

Targetable oncogenic alterations are found in approximately half of lung adenocarcinomas.^29^ Over the past 2 decades, the development of targeted agents has led to specific treatment guidelines.^26,30^ These targeted therapies typically generate high response rates; however, nearly all tumors recur.^30^ Covalent KRAS G12C inhibitors are recent additions to targeted treatments,^30^ but so far with modest benefit due to the diverse and rapidly adapting nature of these cancers.^31^

The effectiveness of immunotherapy in oncogene-driven NSCLC varies: *EGFR-*mutated NSCLC generally do not respond; however, *KRAS*-mutated NSCLC appears to be sensitive.^32^ While combining immunotherapy with targeted therapy usually generates high incidence of adverse events, a study combining adagrasib with pembrolizumab reported a manageable safety profile.^33^

### Antibody-drug conjugates

Antibody-drug conjugates (ADCs) represent an emerging category of therapeutic agents in oncology, blending the precision of targeted therapies with the power of chemotherapy.^34^ Trastuzumab deruxtecan is approved for patients with advanced NSCLC and *HER2* mutations.^34^ Patritumab deruxtecan and telisotuzumab vedotin have been granted FDA Breakthrough Therapy Designation.^35,36^ Datopotamab deruxtecan recently showed a prolonged progression-free survival (PFS) compared to docetaxel.^37^ These findings could potentially reshape the second-line therapy. Other ADCs are being explored, either as monotherapies or in combination.^34^ ADCs come with specific side effects including pulmonary, hepatic, neurological, and ophthalmic.^34^ Optimizing the drug-to-antibody ratio and selecting the appropriate linker type are critical to minimizing these toxicities. Furthermore, the high cost of ADCs poses a significant barrier to global accessibility.

### Newer agents

Novel immunotherapeutic approaches include bi- and trispecific antibodies, T-cell engagers (BiTEs and TriTEs), vaccines, cellular therapies including tumor-infiltrating lymphocytes (TILs), chimeric antigen receptor (CAR)-T cells, natural killer (NK) cells, and neoantigen vaccine-based methods.^38^

Bispecific and trispecific antibodies are designed to engage 2 or 3 distinct epitopes. They work by linking immune cells to tumor cells to increase cytotoxicity, blocking signaling pathways, or targeting immunomodulatory molecules.^39^ Research is also advancing on BiTEs and TriTEs, which redirect immune cells to attack cancer cells, for both small cell lung cancer (SCLC) and NSCLC.^39^

Personalized neoantigen-based cancer vaccines aim to expand the range of tumor-specific immune responses, and guide T cells into tumors.^40^ A variety of platforms are available, including RNA, DNA, protein/peptides, and microbial and cellular vectors, like adenovirus vectors and dendritic cells.^41^

T-cell transfer therapies (including TILs, CAR-T, and NK cell therapies) may be useful for tumors refractory to immunotherapy; however, these therapies face challenges including timing, costs, and manufacturing limitations, and none have yet received regulatory approval.^38^ Increased activity of TILs specific to a broader range of neoepitopes has been linked to better clinical outcomes.^38^ This highlights the need to expand the range of tumor antigens recognized and to enhance the durability and effectiveness of these transferred cells.

### Radiotherapy

Strategies to enhance immune responses by converting “cold” into “hot” microenvironments are gaining attention, with radiotherapy being a potential key player. The proposed mechanism involves increasing antigen release, boosting T-cell and dendritic cell functions, and amplifying the overall anti-tumor immune response.^42^ Additionally, radiotherapy may directly alter the immune microenvironment.^42^ New techniques, including adaptive irradiation to adjust radiation doses and fields during treatment, may reduce toxicity and maintain efficacy.^43^ Current clinical trials are exploring radiotherapy as a consolidation treatment or in combination with chemotherapy and immunotherapy.^44^ Furthermore, trials investigating radiotherapy combined with novel immunotherapies are underway.

### Combination strategies

Combination strategies use immunotherapy with chemotherapy, radiotherapy, or targeted therapy together, potentially leading to synergistic effects for more complete and lasting responses. Combinations may also address points in signaling pathways, helping to prevent or delay drug resistance.

Two recent combinations showed superior PFS in patients with advanced *EGFR*-mutated NSCLC: one combined osimertinib with chemotherapy,^45^ and another amivantamab with lazertinib.^46^ Trials combining immunotherapy and KRAS inhibitors are also in progress.^33^

In situations where a combination of agents proves more effective than single agents of modest impact, collaboration between companies will be vital for comprehensive research. Academic trials, driven by scientific inquiry and open-access data, are ideal for exploring combination treatments. Testing platforms should foster collaborations between academic institutions and companies, while ensuring legal safeguards to protect intellectual property.

Calls to Action are shown in Figure 3.

## Optimizing treatment personalization

Completing comprehensive biomarker testing for non-squamous NSCLC before treatment is essential to guide therapy.^26,30^ While not currently recommended, molecular profiling for squamous NSCLC or SCLC remains under consideration.^26,30^ While next-generation sequencing (NGS) facilitates comprehensive testing,^30^ it is costly and still limited by challenges in reimbursement.^47^ Some genetic alterations (eg, gene fusions) require additional RNA sequencing.^48^ Global testing rates remain low, particularly in underserved communities, and setting up more molecular diagnostic labs, improving data and technology resources, and using molecular tumor boards might increase its deployment.^49^ Genomic testing of circulating cell-free DNA is increasingly used, although less sensitive and comprehensive,^30^ but useful if a biopsy is inconclusive or there is a need to start treatment quickly.

### Biomarkers for immunotherapy

PD-L1 status is a recognized biomarker for ICIs, but with limited predictive accuracy.^16^ Alternative biomarkers including tumor mutational burden, genomic co-mutations, and microsatellite instability have shown inconsistent predictive performances and lack validation.^16,50^ Insights into the tumor microenvironment are ongoing, evaluating the microbiome, transcriptome, epigenetics, and genomic co-mutations.

### On-treatment testing to guide therapy

During treatment, signs of progression may be identified before they appear in radiological exams. On-treatment biopsies may allow an earlier switch in treatment, reducing exposure to ineffective and potentially toxic therapies. However, performing repeated biopsies may exclude patients from clinical trials because of inaccessible tumors or reluctance to undergo invasive procedures. Evaluation of circulating tumor DNA (ctDNA) for tumor response might be more feasible.

In other cancers, particularly hematologic, concepts of minimal residual disease have emerged to guide therapy.^51^ Adapting this approach to lung cancer needs further exploration.

Genomic characterization is key for predicting responses to targeted treatments. Although multiple large datasets exist, access to genomic repositories remains limited. Projects including the Genomics Evidence Neoplasia Information Exchange, a public cancer registry combining clinical and genomic data from top cancer centers, demonstrate the benefit of harmonizing NGS data with clinical outcomes.^52^ Establishing large repositories of DNA and RNA sequencing, along with protein expression and outcome data, and overcoming regulatory barriers to data sharing, both nationally and internationally are essential for progress in this field.

Calls to Action are shown in Figure 3.

## Key topics in SCLC treatment

Over the past decade, the subdivision of SCLC into molecular subtypes and studies on tumor cell plasticity have unveiled the complex nature of this aggressive cancer.^53^ However, a deeper investigation is crucial, as despite high initial response rates, most SCLC relapse within months of chemoimmunotherapy.^53^ Moreover, transformed SCLC, which evolves from adenocarcinoma under the pressure of targeted therapies, remains poorly characterized.^54^ A subset of patients benefit from immunotherapy, and targeting epigenetic factors to restore antigen presentation could further improve treatment responses.^55^ MHC class I is suggested as a biomarker for the efficacy of immunotherapy.^56^ Innovative approaches to increase responses to immunotherapy include MHC-independent bispecific T-cell engagers (BiTEs) and agents targeting DNA damage repair mechanisms.^53^ Tarlatamab, a BiTE targeting delta-like ligand 3 and CD3, recently received FDA accelerated approval for extensive stage SCLC with disease progression on or after platinum-based chemotherapy.^57^ Other ADCs for SCLC are under investigation, with targets including TROP2, B7H3, and mesothelin.^58^

**Figure 4.**
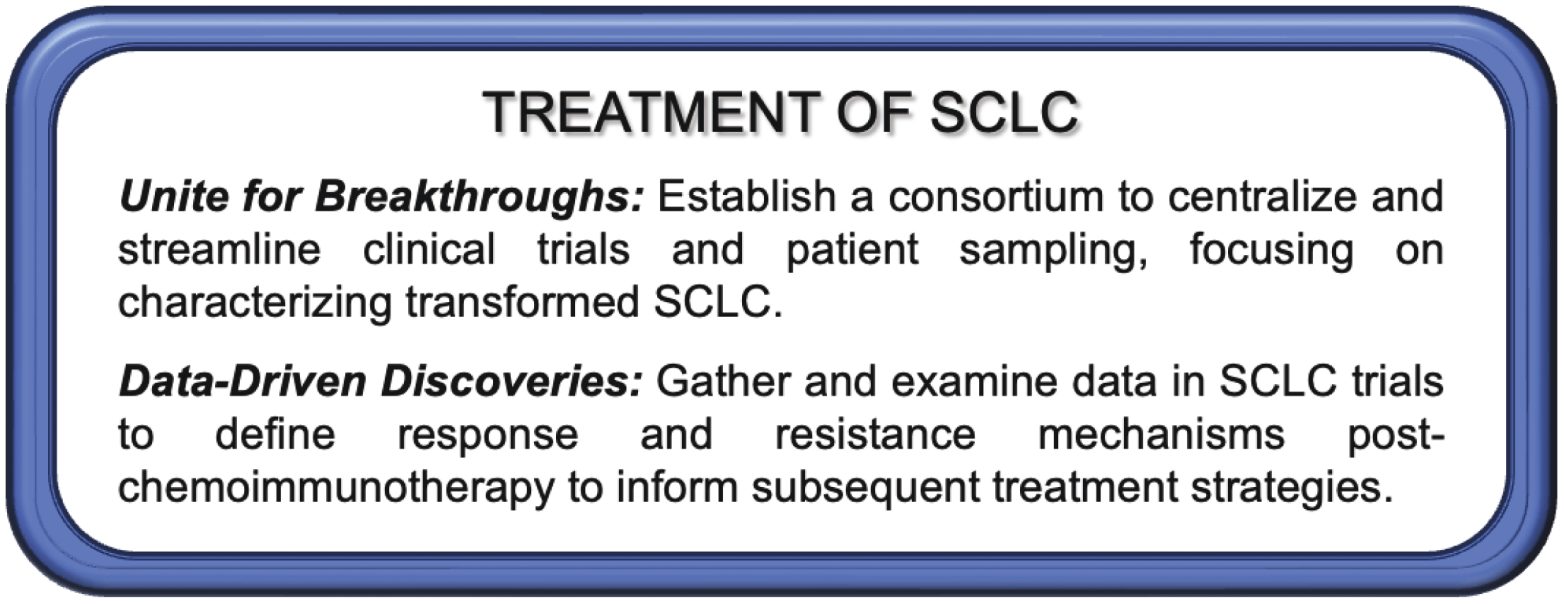
2023 New York Lung Cancer Foundation Consensus Summit Calls to Action—strategies for treatment of SCLC.

Calls to Action are shown in Figure 4.

## New technologies

The use of new technologies for early lung cancer detection is promising. However, if ctDNA is valuable for assessing therapy effectiveness, guiding treatment intensity and duration, and detecting early recurrence or relapse, its current sensitivity and specificity are not adequate for routine early lung cancer detection.^59^ Ongoing studies on multicancer early detection tests analyze DNA methylation patterns to identify potential cancer signals.^60^ Other blood-based techniques for lung cancer detection include fragmentomics,^61^ micro-RNA,^62^ and extra-cellular vesicles.^63^ Although these techniques correlate with recurrence, a major limitation is that negative results do not guarantee the absence of disease. Moreover, plasma-based ctDNA tests do not provide insights into the tumor microenvironment.

The integration of plasma-based biomarker testing with CT screening shows promise; however, it is yet to be determined for which cancer types these methods are more suited.^64^ Technologies such as PhasED-seq, which reduces background NGS errors by tracking multiple mutations on the same gene sequence, could serve as early surrogate endpoints in clinical trials.^65^

Artificial intelligence (AI) is expected to significantly change the roles of pathologists and laboratory technicians in making diagnoses, but regulatory approval processes need to evolve to keep pace. AI research is advancing in lung cancer screening, potentially improving diagnostic accuracy and efficiency,^66^ but diagnostic, legal, and ethical challenges may arise from inaccuracies in predictive algorithms.^67^ This is particularly concerning for minorities, as machine-learning algorithms are developed based on integrated datasets that underreport underserved communities.^67^

**Figure 5.**
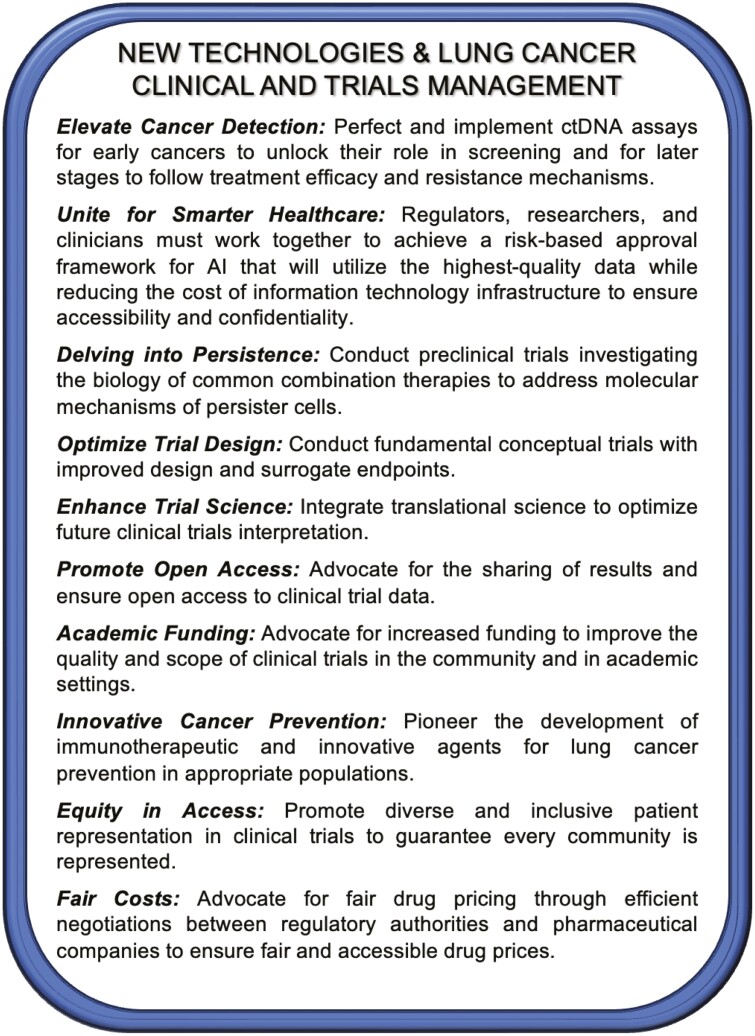
2023 New York Lung Cancer Foundation Consensus Summit Calls to Action—strategies for the use of new technologies and improved clinical and trials management in lung cancer.

Calls to Action for new technologies in lung cancer are shown in Figure 5.

## What are we missing in lung cancer management?

### Innovative prevention approaches

While research has explored various pharmacological agents and dietary interventions for the prevention of lung cancer, none has yet been confirmed as effective.^68^ There is increasing interest in the potential of targeted therapies,^69^ NF-κB inhibitors, and anti-IL-1β monoclonal antibody.^70,71^ Identifying high-risk individuals could be critical for trial designs, incorporating immunomodulatory approaches, such as cancer vaccines or ICIs.

### Combating disease recurrence

Ongoing research suggests that acquired resistance may arise either from the selection of preexisting resistant clones or from cancer cells entering a slowly cycling, senescent state, known as drug-tolerant persisters (DTP) cells.^72^ On-treatment molecular profiling techniques, including NGS, ctDNA, and RNA sequencing (RNA-seq), may elucidate the determinants of resistance mechanisms in the senescent state. Effective interventions at this state may prevent and treat the DTP state. Data on innate resistance and the resistance mechanisms may guide the optimization of treatment strategies leading to better outcomes.

### Improving clinical trial design

Mandatory biopsies and sampling, as well as regulatory demands for central testing, restrict participation to patients treated at large medical centers. Less rigid study requirements may facilitate patient enrollment. Using surrogate endpoints in early-stage perioperative trials with lengthy survival times could hasten regulatory approval, getting new therapies to patients faster.

Prospective analyses before treatment selection have been shown to yield better outcomes compared to unmatched therapy.^73^ Innovative trial designs might include initial treatment followed by on-treatment biopsies or ctDNA assessments, with adaptive randomization to treatments based on real-time integrated biomarker analyses.

Open access to data and shared results of industry-sponsored trials should be guaranteed. Additionally, there is widespread agreement on the necessity of increasing funding for academic and community-based research.

Ensuring broad patient representation in clinical trials is a critical step toward improved care including increased access to clinical trials in countries around the world, accompanied by heightened educational initiatives. Conducting global multicountry clinical trials with diverse populations and accounting for varied genetic profiles is essential to define potential differences in outcomes across the world. It is vital to conduct these trials ethically and ensure post-trial treatment availability, especially when evaluating surrogate endpoints.

### Address health disparities in drug access and cost

Several factors limit access to drugs, including economic constraints, legal and policy barriers, insufficient awareness, social behavior, and fragmented knowledge.^74^ Implementing a pricing policy that promotes affordable drugs is essential and should be integrated into national drug policies.^74^ Potential strategies might involve tiered pricing, limited development costs, and production of biosimilars.

Calls to Action are shown in Figure 5.

## Conclusion: current priorities in lung cancer research

**Figure 6.**
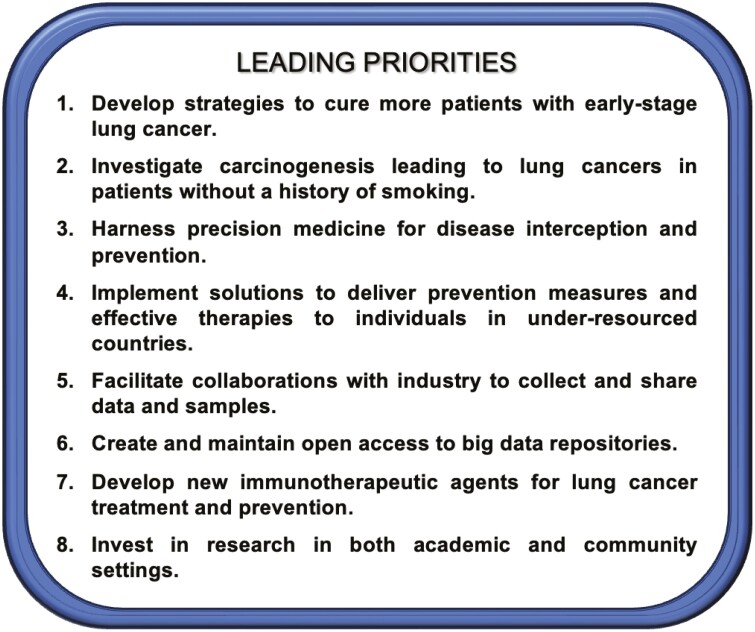
The leading priorities determined by the 2023 New York Lung Cancer Foundation Consensus Summit.

Panelists from the New York Lung Cancer Foundation (NYLCF) identified 8 key priorities that warrant immediate attention (Figure 6). Strengthening partnerships across industries and engaging nonacademic stakeholders could streamline research progress and ensure the inclusion of diverse patient subgroups in clinical trials, leading to more comprehensive and effective lung cancer treatments.

## Data Availability

No new data were generated or analysed in support of this research.
